# Trabecular bone scores in children with osteogenesis imperfecta respond differently to bisphosphonate treatment depending on disease severity

**DOI:** 10.3389/fped.2024.1500023

**Published:** 2024-12-03

**Authors:** Natsuko Futagawa, Kosei Hasegawa, Hiroyuki Miyahara, Hiroyuki Tanaka, Hirokazu Tsukahara

**Affiliations:** ^1^ Department of Pediatrics, Okayama University Graduate School of Medicine, Dentistry and Pharmaceutical Sciences, Okayama, Japan; ^2^ Department of Pediatrics, Okayama University Hospital, Okayama, Japan; ^3^ Department of Pediatrics, Okayama Saiseikai General Hospital, Okayama, Japan

**Keywords:** bone density, osteoporosis, bone diseases, connective tissue, child

## Abstract

**Introduction:**

Osteogenesis imperfecta (OI) is a congenital skeletal disorder characterized by bone fragility. Bisphosphonates (BISs) have become the mainstream treatment in children with OI. However, an optimal treatment protocol has not yet been established, while BIS treatment tends to be administered to normalize bone mineral density (BMD). Bone quality is an important component of bone strength. The trabecular bone score (TBS) is a quantitative measure of the microstructure that affects bone quality. This study investigated the TBS during BIS treatment in children with OI.

**Materials and methods:**

Twenty-nine children with OI were enrolled and classified into two groups: mild (type 1) and moderate to severe (types 3 and 4). Dual-energy x-ray absorptiometry images were retrospectively analyzed for TBS calculation. The relationship between the areal BMD (aBMD), its Z-score, height-adjusted BMD (BMD_HAZ_) Z-score, TBS, and TBS Z-score with the treatment duration was assessed for each group.

**Results:**

In the mild group, the aBMD, its Z-score, and BMDHAZ Z-score showed a significant positive correlation with treatment duration (r = 0.68, 0.68, 0.72, respectively, *p* < 0.01). The TBS Z-score tended to increase with treatment duration, albeit without reaching significance. In the moderate to severe group, the TBS Z-score showed a significant positive correlation with treatment duration (r = 0.48, *p* < 0.01), in contrast to the aBMD Z-score, which did not increase. Finally, the BMDHAZ Z-score only showed a weak positive correlation with treatment duration (r = 0.37, *p* < 0.01).

**Conclusion:**

Because BIS affect the BMD and TBS differently based on the severity of OI, treatment goals may need to be stratified by disease severity.

## 1 Introduction

Osteogenesis imperfecta (OI) is a congenital skeletal disorder characterized by varying degrees of bone fragility and extraskeletal manifestations, such as dentinogenesis imperfecta and blue sclera. OI has an estimated incidence of 1 out of 15,000–20,000 individuals, and approximately 90% of patients display pathogenic variants in the *COL1A1* and *COL1A2* genes, which encode the *α*1 and *α*2 chains of type I collagen, respectively. Other than *COL1A1* and *COL1A2*, 20 causative genes have recently been identified (1).

In 1979, Sillence et al. developed a clinical classification that categorizes OI into types I to IV according to clinical manifestations, such as bone deformity, blue sclera, dentinogenesis imperfecta, and mode of inheritance (2)*.* Type I OI shows a mild clinical phenotype, and is characterized by bone fragility without bone deformities, as well as mild growth retardation. Type II OI displays the most severe phenotype and is characterized by multiple fractures during the fetal period and respiratory impairment due to a hypoplastic thorax and multiple rib fractures. Type III OI exhibits a severe clinical phenotype, and is characterized by progressive bone deformity, severe growth retardation, and high bone fragility. Type IV OI is of intermediate clinical severity between types I and III. Recently, a modified classification system that categorizes OI into types 1 to 5 has been developed, adding type 5 OI to the classical classification (3); type 5 OI is characterized by calcification of interosseous membranes and hyperplastic callus caused by recurrent c.-14C > T variant in *IFITM5* (4, 5).

Bisphosphonates (BISs) have become the mainstream treatment for bone fragility in children with OI. BISs increase bone mineral density (BMD) by inhibiting bone resorption (6, 7). Nevertheless, an optimal treatment protocol and duration have not yet been established for children with OI. In Japan, the cyclic pamidronate infusion reported by Rauch (8) is frequently used, and the goal of treatment is often set to normalize the BMD.

Bone strength is defined not only by BMD, but also bone quality. On the one hand, BMD accounts for 70% of bone strength (9) and is often measured using dual-energy x-ray absorptiometry (DXA). In children, BMD is influenced by age, pubertal stage (10), and height, with individuals with short stature tending to exhibit lower areal BMD (aBMD) because of their relatively small vertebrae (11). On the other hand, bone quality, the remaining component of bone strength, is influenced by bone microstructure, bone turnover, microfractures, and bone tissue calcification.

Recently, the trabecular bone score (TBS) has been developed as a method of evaluating the bone microstructure (12). The TBS provides an indirect index of the trabecular microarchitecture, which cannot be obtained from BMD alone. Because the TBS represents the connections of the trabecular bone, a higher TBS indicates dense bone trabecular connections. Previous studies used the TBS with BMD to estimate bone fragility in various conditions, such as type 2 diabetes mellitus in adults, postmenopausal osteoporosis, and anorexia nervosa (13, 14). Similar to BMD, the TBS is affected by children's age and pubertal status. In particular, the TBS in healthy children aged 10–18 years increases with age and pubertal maturation (15).

Abundant evidence suggests that BIS treatment increases BMD in patients with OI; however, evidence for bone fracture prevention is limited (16). Some reports have pointed out that long-term BIS treatment may be a risk factor for atypical fractures, characterized by increased bone fragility despite the increase in BMD and cortical thickness (17), as excessive BIS treatment can still decrease bone quality and increase bone fragility (18, 19). Therefore, evaluation of both BMD and bone quality is necessary to determine the efficacy of BIS treatment and to assess the fracture risk in children with OI. However, methods of bone quality evaluation during BIS treatment in children with OI have not yet been established.

This study aimed to analyze the TBS during BIS treatment in children with OI, and to investigate the relationship between the TBS and age and treatment duration based on disease severity. We speculated that BISs would not only increase BMD but would also affect trabecular bone formation and that the duration of BIS treatment would correlate with the TBS since BISs inhibit bone resorption. We also hypothesized that the change of TBS during BIS treatment would differ based on disease severity because previous reports have shown that atypical femur fracture is related to disease severity, not to treatment duration (20).

## 2 Materials and methods

### 2.1 Study population

A total of 29 Japanese children with OI were retrospectively enrolled in this study and classified into two groups: mild (type 1 OI) and moderate to severe (type 3 and 4 OI). Data on medical history, clinical classification, fracture history, and BIS treatment were collected from the medical records. No patients had any history of spinal operations for scoliosis. Z-scores for the height and body mass index of children with OI were calculated using a calculation tool for the growth evaluation of Japanese children (21). Disease-causing genetic variants were obtained from our previous study (22). Based on the patient's medical record, puberty was defined as a testicular volume greater than 4 ml in males and breast development greater than Tanner stage 2 in females.

### 2.2 Treatment protocol

All children with OI were treated using either of the following three protocols: intravenous pamidronate only (PAM), oral alendronate only (ALN), and switching from PAM to ALN (PAM to ALN). PAM was infused cyclically based on a previous report (8). ALN treatment was administered before puberty; a 35-mg tablet was taken once every two weeks and twice a month, while after puberty, a 35-mg tablet was taken thrice a month. PAM was switched to ALN when the patients could swallow a 35-mg ALN tablet.

### 2.3 Measurement method for BMD and TBS

DXA images performed at Okayama University Hospital from April 1, 2006, to March 31, 2017, were retrospectively analyzed. The aBMD (L1–L4) was measured using Discovery A (Hologic Inc., Massachusetts, USA). DXA images of the lumbar spine (L1–L4) were examined for TBS calculation using TBS iNsight (Medimaps SA, France, ver3.0.2.0). The aBMD Z-score of the participants was obtained at >1 year of age, as standard data from healthy children are only available from the age of 1 year. The height-adjusted BMD (BMD_HAZ_) Z-score was calculated using the BMD Z-score and data from previous studies in healthy children (23).

The TBS values obtained from L1 to L4 were averaged and used. As for the calculation of the TBS Z-score, the average and standard deviation (SD) of the TBS in healthy children from a previous study were used (14). Because the average and SD of the TBS in healthy children were only available from ≥5 years of age, the TBS Z-score of the participants after ≥5 years of age could also be obtained.

### 2.4 Statistical analysis

Differences between two independent groups were examined using the Mann–Whitney *U*-test, and the correlation between two factors was analyzed using Spearman's rank correlation coefficient. All statistical analyses were performed using EZR software, with statistical significance set at *p* < 0.05 (24).

### 2.5 Ethical considerations

All procedures in this study were performed in accordance with the 1964 Declaration of Helsinki, as well as the 2003 Ethical Guidelines for Clinical Research and later amendments. This study was approved by the Ethics Committee of Okayama University Graduate School of Medicine, Dentistry and Pharmaceutical Sciences and Okayama University Hospital (1809–002). Informed consent was obtained in the form of opt-out on the website.

## 3 Results

### 3.1 Baseline characteristics

Table 1 summarizes the clinical characteristics of patients at their first DXA scan at our hospital. Overall, 17 and 12 patients with OI were classified into the mild and moderate to severe groups, respectively. The mean height Z-score was significantly lower in the moderate to severe group than in the mild group. The mean number of DXA measurements for all patients with OI was 5.7 (1–15) (Supplementary Table S1). The interval between the first and last DXA measurements was significantly longer in the moderate to severe group than in the mild group (The average interval was 3.3 years and 6.3 years, respectively).

**Table 1:** Clinical characteristics of 29 children with OI at the first DXA measurement.

		All			Mild group			Moderate to severe group		
		Total	Male	Female	Total	Male	Female	Total	Male	Female
Number of patients (Number of pubertal patients: age of pubertal beginning)		29 (13:10.4 ± 0.84)	15 (5: 11.0 ± 0.58)	14 (8:10.0 ± 0.74)	17 (8:10.4 ± 0.92)	10 (5: 11.0 ± 0.58)	7 (3: 9.4 ± 0.42)	12 (5: 10.3 ± 0.70)	5 (0: -)	7 (5: 10.3 ± 0.70)
Age (years)		5.4 (0.1–14.3)	6.9 (0.1–14.3)	3.6 (0.1–9.0)	6.2 (0.1–14.3)	7.6 (0.1–14.3)	4.2 (0.7–9.0)	4.2 (0.1–13.0)	5.5 (0.3–13.0)	3.3 (0.1–5.7)
BIS treatment	PAM	7	3	4	4	2	2	3	1	2
	ALN	6	5	1	6	5	1	0	0	0
	PAM→ALN	13	5	8	5	2	3	8	3	5
	Before treatment	3	2	1	2	1	1	1	1	0
Height (cm)		96.9 (48.3–149.4)	106.7 (48.3–149.4)	86.4 (49.4–121.5)	105.1 (55.0–149.4)	113.1 (55.0–149.4)	93.6 (70.5–121.5)	85.3 (48.3–129.4)	93.8 (48.3–129.4)	79.3 (49.4–98.0)
Height Z-score		−1.97 ± 1.65	−1.72 ± 1.85	−2.23 ± 1.35	−1.20 ± 1.13	−1.01 ± 1.19	−1.47 ± 0.97	−3.05 ± 1.65**	−3.13 ± 2.10*	−3.00 ± 1.24*
Weight (kg)		17.4 (4.0–42.6)	22.3 (4.4–42.6)	12.2 (4.0–30.7)	20.9 (4.4–42.6)	25.3 (4.4–42.6)	14.8 (7.3–30.7)	12.5 (4.0–33.9)	16.4 (4.5–33.9)	9.6 (4.0–13.3)
BMI Z-score		−0.14 ± 1.29	−0.10 ± 1.10	−0.18 ± 1.46	−0.13 ± 1.27	−0.45 ± 0.88	−0.19 ± 1.44	−0.14 ± 1.32	−0.10 ± 1.07	−0.17 ± 1.47
Mean number of DXA measurements		5.7 (1–15)	4.7 (1–14)	6.9 (1–15)	5.2 (1–14)	5.0 (1–14)	5.6 (1–11)	6.4 (1–15)	4.0 (1–9)	8.1 (5–15)
The interval between the first and last DXA measurement (years)		4.6 (0.0–10.0)	3.7 (0.0–9.7)	5.5 (0.0–10.0)	3.3 (0.0–7.8)	3.3 (0.0–7.8)	3.4 (0.0–5.8)	6.3* (0.0–10.1)	4.6 (0.0–9.7)	7.5 (4.0–10.1)

Age, height, and weight are presented as mean (range). Height and BMI Z-scores are shown as mean ± standard deviation.

* Significantly lower than that in the mild group (*p* < 0.05). ** Significantly lower than that in the total mild group (*p* < 0.01).

OI, osteogenesis imperfecta; DXA, dual-energy x-ray absorptiometry; BMI, body mass index; ALN, oral alendronate only; PAM, intravenous pamidronate only; BIS, bisphosphonates.

### 3.2 Genetic background of children with Oi

Table 2 shows the genetic background of patients with OI enrolled in this study. Genetic analysis was conducted on 23 out of 29 patients, which identified pathogenic genetic variants in *COL1A1* and *COL1A2* in all 23 analyzed patients (22). Mild group patients exhibited quantitative defects, including nonsense, splicing defects, and frameshift mutations in *COL1A1*, except for one patient with a missense variant of *COL1A1.* In contrast, moderate to severe group patients displayed qualitative defects, including glycine (Gly) substitutions in the Gly-X-Y repeat with other amino acids in *COL1A1* and *COL1A2*, except for one patient with three-amino acid deletion of *COL1A1* and one patient with a frameshift in *COL1A2*.

**Table 2:** Genetic aberrations identified in the 29 children with OI.

		All (*n* = 29)		Mild (*n* = 17)		Moderate to severe (*n* = 12)	
		*COL1A1*	*COL1A2*	*COL1A1*	*COL1A2*	*COL1A1*	*COL1A2*
Types of variant	Nonsense	2	0	2	0	0	0
	Splicing defect	6	1	6	1	0	0
	Frameshift	4	1	4	0	0	1
	Missense	3	5	1	0	2	5
	Three-amino acid deletion	1	0	0	0	1	0
	Unknown	6		3		3	

OI, osteogenesis imperfecta.

### 3.3 Correlation of aBMD and TBS with patients' age

**Figure 1:**
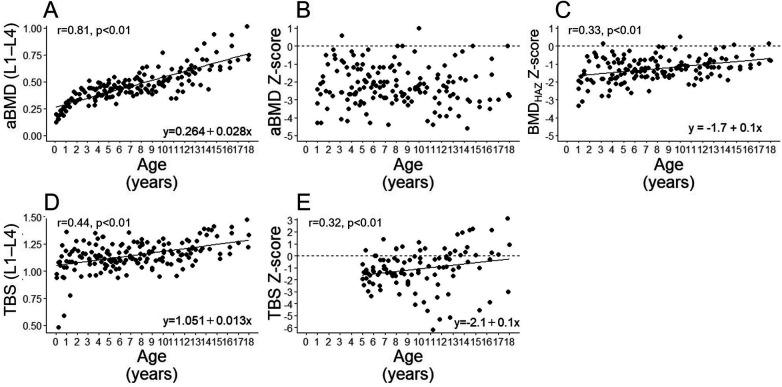
Correlations of age with the aBMD (L1–L4) **(A)**, aBMD Z-score **(B)**, BMD_HAZ_ Z-score **(C)** TBS (L1–L4) **(D)**, and TBS Z-score **(E)** in all participants. aBMD, areal bone mineral density; BMDHAZ, height-adjusted bone mineral density.

Figure 1 shows the correlation between the aBMD (L1–L4), its Z-score, BMD_HAZ_ Z-score, TBS (L1–L4), and TBS Z-scores with the patients' age during the study period. The aBMD (L1–L4) showed a significant positive correlation with age (Figure 1A), and its Z-score was almost distributed below 0 and was not correlated with age (Figure 1B). In contrast to aBMD (L1–L4), the aBMD Z-score did not correlate with age, but the BMD_HAZ_ Z-score showed weak positive correlation with age (Figure 1C).

The TBS (L1–L4) and its Z-score were positively correlated with age (Figures 1D,E, respectively).

### 3.4 Correlation of the aBMD Z-score and TBS Z-score with treatment duration

**Figure 2:**
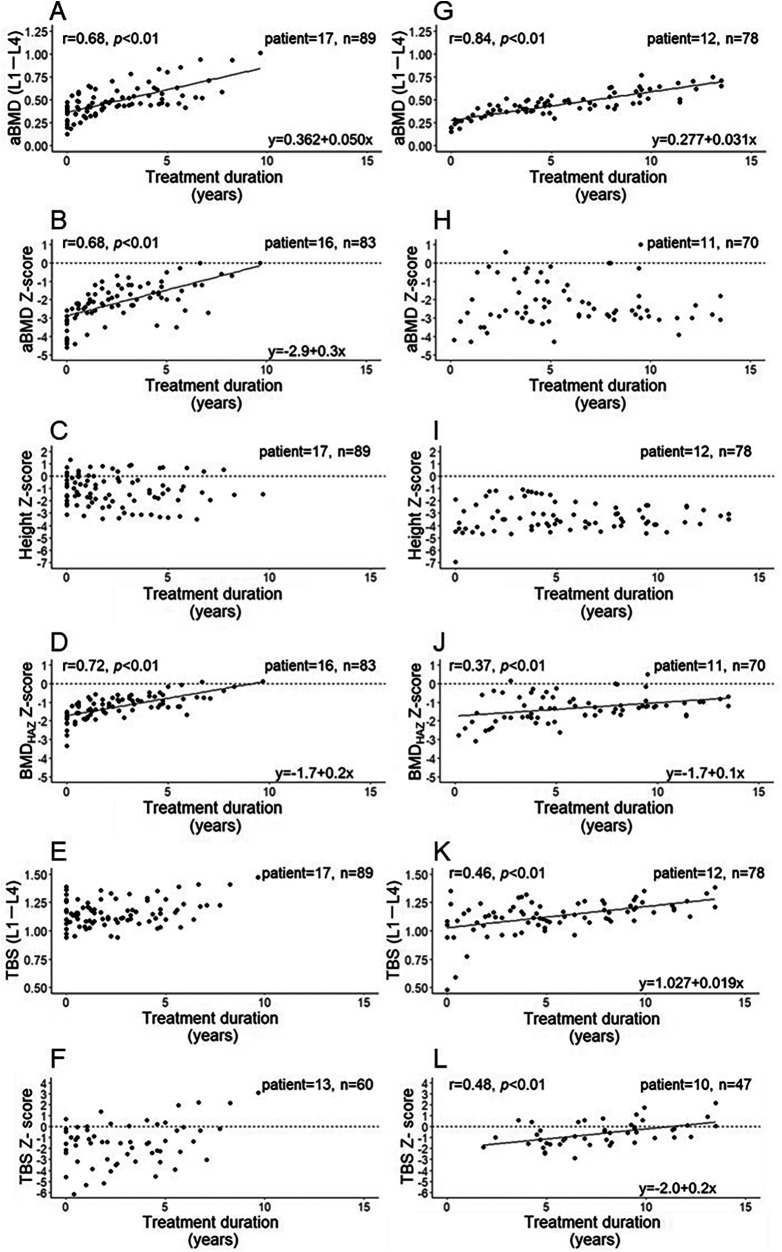
Correlation of BIS treatment duration with the aBMD (L1–L4), aBMD Z-score, height Z-score, BMD_HAZ_ Z-score, TBS (L1–L4), and TBS Z-score in the mild **(A** to **F**, respectively) and moderate to severe groups **(G** to **L**, respectively). aBMD, areal bone mineral density; N, number of patients; n, number of measurements.

Next, the relationships between treatment duration and aBMD (L1–L4), aBMD Z-score, height Z-score of patients, BMD_HAZ_ Z-score, TBS (L1–L4), and TBS Z-score were explored in the mild (Figures 2A–F) and moderate to severe groups (Figures 2G–L), as we had hypothesized that an increase in TBS would differ based on disease severity. The transition of bone parameters in each patient and the transition of the height of the patients with OI are shown in Supplementary Figures S1–S6. In the mild group, the aBMD (Figure 2A) and its Z-score (Figure 2B) were significantly positively correlated with treatment duration. Conversely, the height Z-score, which affects aBMD and its Z-score, was not correlated with age (Figure 2C). However, after adjusting the BMD Z-score for patients' height, the BMD_HAZ_ Z-score was still significantly positively correlated with treatment duration (Figure 2D). Furthermore, the TBS (Figure 2E) and its Z-score (Figure 2F) tended to increase as the treatment duration increased; however, the correlation with treatment duration was not significant.

In the moderate to severe group, the aBMD showed a significant positive correlation with treatment duration (Figure 2G), whereas the aBMD Z-score did not increase with longer treatment duration; a correlation with treatment duration was also not observed (Figure 2H). During the treatment periods, the height Z-score was significantly lower in the moderate to severe group (Figure 2I) than in the mild group (Figure 2C) (*p* < 0.01), and did not correlate with the treatment duration. As with the mild group, the BMD_HAZ_ Z-score was significantly positively correlated with treatment duration in the moderate to severe group; however, this correlation was weaker than that in the mild group (Figure 2J). The TBS and its Z-score were significantly positively correlated with treatment duration (Figures 2K,L, respectively).

### 3.5 Comparison of parameters at the first and the last DXA measurements after 5 years old by severity

To examine the difference in the change of BMD and TBS in each of the severity groups, 21 patients whose BMD was measured at least twice after 5 years were analyzed. Table 3 shows the change of the aBMD Z-score, BMD_HAZ_ Z-score, and TBS Z-score from the first to the last DXA measurement after 5 years of age, by severity. Age at the final DXA measurement showed no significant difference between the mild and moderate to severe groups. The duration of the first to the last TBS measurement was significantly longer in the moderate to severe group than in the mild group. In both groups, no significant increase in the height Z-score was observed, while the height Z-score of the moderate to severe group was consistently lower than that of the mild group. In the mild group, the aBMD Z-score increased more than that in the moderate to severe group, and the change in the aBMD Z-score per year was significantly larger than that in the moderate to severe group. This tendency was observed in the BMD_HAZ_ Z-score. However, the TBS (L1–L4) and TBS Z-score at the final DXA measurement were found to be significantly higher in the moderate to severe group than in the mild group. The change in the TBS Z-score per year in the moderate to severe group tended to be higher than that in the mild group, although the difference was not significant.

**Table 3:** Comparison of BMD and TBS between the first and the last measurements after the age of 5 years by severity.

		Mild	Moderate to severe
Number of patients (male/female)		12 (7/5)	9 (3/6)
Number of pubertal patients (male/female)		7 (5/2)	5 (0/5)
BIS treatment	PAM	2	2
	ALN	5	0
	PAM→ALN	5	7
Age (years)		6.7 ± 3.9/11.7 ± 3.7	1.7 ± 1.8*/12.1 ± 2.8
Interval		3.3 ± 1.7	5.6 ± 2.4*
Height Z-score		−1.1 ± 1.1/−1.1 ± 1.2	−3.2 ± 1.1**/−3.2 ± 0.9**
aBMD (L1-L4)		0.44 ± 0.09/0.65 ± 0.14	0.43 ± 0.08/0.62 ± 0.11
aBMD Z-score		−2.78 ± 0.98/−1.38 ± 0.65	−2.50 ± 1.18/−1.92 ± 1.30
ΔaBMD Z-score/year		0.58 ± 0.47	0.16 ± 0.22*
BMD_HAZ_ Z-score		−1.57 ± 0.49/−0.62 ± 0.27	−1.53 ± 0.69/−0.82 ± 0.59
ΔBMD_HAZ_ Z-score/year		0.37 ± 0.25	0.16 ± 0.13*
TBS (L1-L4)		1.16 ± 0.09/1.20 ± 0.11	1.13 ± 0.09/1.27 ± 0.08
TBS Z-score		−1.38 ± 1.43/−1.25 ± 1.75	−1.16 ± 1.01/0.54 ± 1.05*
ΔTBS Z-score/year		0.05 ± 0.52	0.20 ± 0.40

Age, interval, Height Z-score, aBMD (L1–L4), TBS (L1–L4), each Z-score, and BMD_HAZ_ Z-score are presented as mean ± SD. These parameters show “the first measurement data/the last measurement data” except for age is shown as “age at the start of BIS treatment/age at the last measurement.” *Δ* in each parameter indicates the difference between the first and the last measurement. *Δ*aBMD Z-score/year, *Δ*BMD_HAZ_ Z-score/year and *Δ*TBS Z-score/year show the *Δ* for each parameter divided by the interval. These parameters are also presented as mean ± SD.

BIS, bisphosphonate; PAM, pamidronate; ALN, alendronate; aBMD, areal BMD; TBS, trabecular bone score; BMD_HAZ_ Z-score, height adjusted BMD Z-score.

* Significant difference compared with than in the mild group (*p* < 0.05). **Significant difference compared with that in the mild group (*p* < 0.01).

## 4 Discussion

The present study analyzed the change in the TBS, aBMD, and height adjusted BMD during BIS treatment in children with OI, clarifying that the increase of TBS during the BIS treatment differs according to the severity of OI. BIS treatment has become the mainstream treatment for patients with OI; various studies have reported that BIS treatment increases BMD. However, the effect of BIS treatment on the TBS according to disease severity is still controversial. Some studies have shown an increase in the TBS by BIS treatment in adult osteoporosis (25); however, few studies have investigated the TBS in patients with OI during BIS treatment.

One study of pediatric patients with OI reported that 1 year of BIS treatment increased the TBS (L1–L4) by 2.1%, but no significant difference in the TBS (L1–L4) was observed before and after BIS treatment; however, the difference by severity was not analyzed (26). In our study, we could not evaluate the TBS in patients younger than 5 years of age owing to the lack of TBS data in healthy children of this age. However, previous studies analyzing bone histomorphometry by bone biopsy in children with OI reported that the trabecular bone number was decreased in the moderate to severe group (types 3 and 4) compared with the mild group (type 1) (27). Another study also reported that the trabecular bone was sparse in the severe group using high-resolution peripheral quantitative computed tomography and that the TBS (L1–L4) in adult patients with OI tended to be lower in the severe group than in the mild group (28). Therefore, under the untreated state, the trabecular bone is sparser, and the TBS is assumed to be lower in the moderate to severe group than in the mild group.

The first TBS Z-score measured for the first time showed no significant difference between the mild and moderate to severe groups (*p* = 1.00), only inpatients 5 years old or older. This could be because the BIS treatment was started at a younger age in the moderate to severe group than in the mild group. It is expected that the TBS had already increased before the 5 years of age when the TBS Z-score could be calculated.

At the final measurement during the study period, the TBS Z-score was significantly higher in the moderate to severe group than in the mild group, and this difference may partly be attributed to the significantly longer treatment duration in the moderate to severe group than in the mild group. In our study, the treatment duration in the moderate to severe group was approximately twice as long as that in the mild group. Another reason for the higher TBS Z-score in the moderate to severe group is a difference in the change rate of the TBS Z-score per year by disease severity, and the TBS Z-score tend to be higher in the moderate to severe group than in the mild group, although the detailed mechanism for this is unknown.

Our study also clarifies that the severity of OI affects the increase of the BMD. In the moderate to severe group, it was difficult to increase the aBMD Z-score with BIS treatment in contrast to the mild group in which the aBMD Z-score significantly increased despite the shorter treatment periods. BMD_HAZ_, the BMD corrected by patient height, showed a positive correlation in the moderate to severe group to the treatment periods, although its correlation is only mild compared with that in the mild group. The BMD_HAZ_ Z-score can represent the true BMD Z-score like the volumetric BMD Z-score. However, the BMD_HAZ_ Z-score, which requires healthy control data in each race to calculate the same value as volumetric BMD, is difficult to use clinically; as such, the aBMD Z-score is commonly used in clinical situations. If treatment is provided for a long time to normalize the aBMD Z-score in the moderate to severe group, the TBS Z-score may be excessively high. Thus, normalization of the aBMD Z-score may not be appropriate as a goal of BIS treatment, particularly in the moderate to severe group.

As the complication of BIS treatment, atypical femoral fracture is well known; however, the relationship between BIS treatment and atypical femoral fractures remains controversial. A previous report showed that prolonged BIS treatment may be a risk factor for atypical femoral fractures; thus, a drug holiday is recommended (29). While it is unknown whether an increased TBS leads to bone fragility, an excessively increased TBS indicates excessively dense trabecular bone and might lead to atypical femur fractures or BIS-induced osteopetrosis (30, 31). Our results may also support another previous report that showed that the severe group itself is related to atypical femoral fractures during BIS treatment in OI, not to BIS treatment duration because patients in the severe group tend to be treated with BIS for longer periods than patients in the mild group (19). Hence, it may be necessary to reconsider the protocol of BIS treatment for children with OI according to the severity of OI.

This study has some limitations. First, as previously mentioned, we could not evaluate the TBS Z-score in patients aged <5 years and the TBS before BIS treatment in the moderate to severe group in contrast to the mild group, in which BIS treatment was started from an older age. Second, our study population comprised only Japanese patients whose stature and pubertal timing are different from those of healthy Caucasians, for which standard data are used for calculating Z-scores of the aBMD, BMD_HAZ_, and TBS. Third, we could not examine the relationship between the frequency of bone fractures and the TBS Z-score during BIS treatment because bone fracture is suppressed by BIS treatment (data not shown). Additionally, we could not consider the treatment interruption periods by bone fracture. However, bone fractures were suppressed by BIS treatment in both severity groups, and the effect of treatment interruption to the TBS and BMD increase by bone fractures was believed to be minimal. In addition, there were cases of lumbar spine fracture, there were no cases of lumbar spine fracture at the time of the DXA scan. Fourth, the participants were treated following different protocols such as PAM, ALN, and PAM to ALN, and these variations could lead to bias. However, since this was a retrospective study, it was not possible to adjust for treatment methods. Finally, as this was a retrospective study with a small sample size, a prospective study with a large cohort is required to clarify the optimal TBS and BMD values for achieving the sufficient reduction of bone fractures in respective OI severities.

## 5 Conclusions

In conclusion, BIS treatment increases the TBS, particularly in patients with moderate to severe OI compared with those with mild OI. While the optimal TBS value for preventing bone fractures in OI remains unknown, we believe that, in combination with BMD measurements, the TBS may serve as an indicator for setting appropriate protocols and goals for BIS treatment in children with OI based on the disease severity.

## Data Availability

Datasets are available on request: The raw data supporting the conclusions of this article will be made available by the authors, without undue reservation.
