# Supplementary material for: Trabecular bone scores in children with osteogenesis imperfecta respond differently to bisphosphonate treatment depending on disease severity
Source: Front Pediatr. 2024 Dec 3;12:1500023. doi: 10.3389/fped.2024.1500023 (PMC11653183; doi:10.3389/fped.2024.1500023)
Supplement: Supplementary file 1 [file Table1.docx]

Supplementary Material

# Supplementary Table: Number of BMD and TBS measurements and the corresponding Z-scores.

| Patient | Severity | All | BMD | BMD Z-score | TBS | TBS Z-score | Gene | Genotype | |
| --- | --- | --- | --- | --- | --- | --- | --- | --- | --- |
| A | Mild | 8 | 8 | 8 | 8 | 5 | COL1A1 |  | c.3207+1G>A |
| B | Mild | 2 | 2 | 1 | 2 | 0 | COL1A1 |  | c.3207+1G>A |
| C | MS | 9 | 9 | 9 | 9 | 7 |  |  |  |
| D | Mild | 6 | 6 | 6 | 6 | 6 | COL1A1 |  | c.1669-1G>A |
| E | MS | 2 | 2 | 2 | 2 | 2 | COL1A1 | p.Gly353Ser | c.1057G>A |
| F | MS | 5 | 5 | 5 | 5 | 5 | COL1A1 | 3aa deletion(Gly Pro Ala) | c.3145_3153del |
| G | MS | 15 | 15 | 11 | 15 | 4 | COL1A2 | p.586insAla | c.1755_1756insGCT |
| H | MS | 5 | 5 | 5 | 5 | 5 | COL1A2 | p.Gly1012Ser | c.3034G>A |
| I | MS | 8 | 8 | 6 | 8 | 0 | COL1A2 | p.Gly1102Val | c.3305G>T |
| J | Mild | 5 | 5 | 5 | 5 | 4 |  |  |  |
| K | MS | 8 | 8 | 8 | 8 | 6 |  |  |  |
| L | Mild | 4 | 4 | 4 | 4 | 4 | COL1A1 |  | c.3207+1_3207+2del |
| M | Mild | 1 | 1 | 1 | 1 | 0 | COL1A1 |  | c.1704del |
| N | Mild | 1 | 1 | 1 | 1 | 0 | COL1A1 |  | c.1704del |
| O | Mild | 14 | 14 | 10 | 14 | 3 | COL1A1 | p.Ala1443Val | c.4328C>T |
| P | MS | 6 | 6 | 6 | 6 | 6 |  |  |  |
| Q | MS | 2 | 2 | 2 | 2 | 1 | COL1A2 | p.Gly328Ser | c.982G>A |
| R | MS | 9 | 9 | 8 | 9 | 4 | COL1A1 | p.Ala1375Thr | c.4123G>A |
| S | MS | 8 | 8 | 8 | 8 | 7 | COL1A2 | p.Gly1012Ser | c.3034G>A |
| T | Mild | 1 | 1 | 0 | 1 | 0 | COL1A1 |  | c.1299+1G>T |
| U | MS | 1 | 1 | 0 | 1 | 0 | COL1A2 | p.Gly358Ser | c.1072G>A |
| V | Mild | 11 | 11 | 11 | 11 | 5 | COL1A1 |  | c.2444del |
| W | Mild | 6 | 6 | 6 | 6 | 6 | COL1A1 | p.Arg882Ter | c.2644C>T |
| X | Mild | 1 | 1 | 1 | 1 | 1 | COL1A2 |  | c.486+1G>A |
| Y | Mild | 4 | 4 | 4 | 4 | 4 |  |  |  |
| Z | Mild | 5 | 5 | 5 | 5 | 5 |  |  |  |
| AA | Mild | 7 | 7 | 7 | 7 | 7 |  |  |  |
| AB | Mild | 6 | 6 | 6 | 6 | 6 | COL1A1 | p.Gly424AlafsTer117 | c.1269del |
| AC | Mild | 7 | 7 | 7 | 7 | 4 | COL1A1 | p.Gln1240Ter | c.3718C>T |
| Total | | 167 | 167 | 153 | 167 | 107 |  |  |  |
| mean | | 5.7 | 5.7 | 5.2 | 5.7 | 3.6 |  |  |  |

MS, moderate to severe; BMD, bone mineral density; TBS, trabecular bone score; 3aa, three amino acids.
