# Supplementary material for: Trabecular bone scores in children with osteogenesis imperfecta respond differently to bisphosphonate treatment depending on disease severity
Source: Front Pediatr. 2024 Dec 3;12:1500023. doi: 10.3389/fped.2024.1500023 (PMC11653183; doi:10.3389/fped.2024.1500023)
Supplement: Supplementary file 2 [file Presentation1.pptx]

## Slide 1
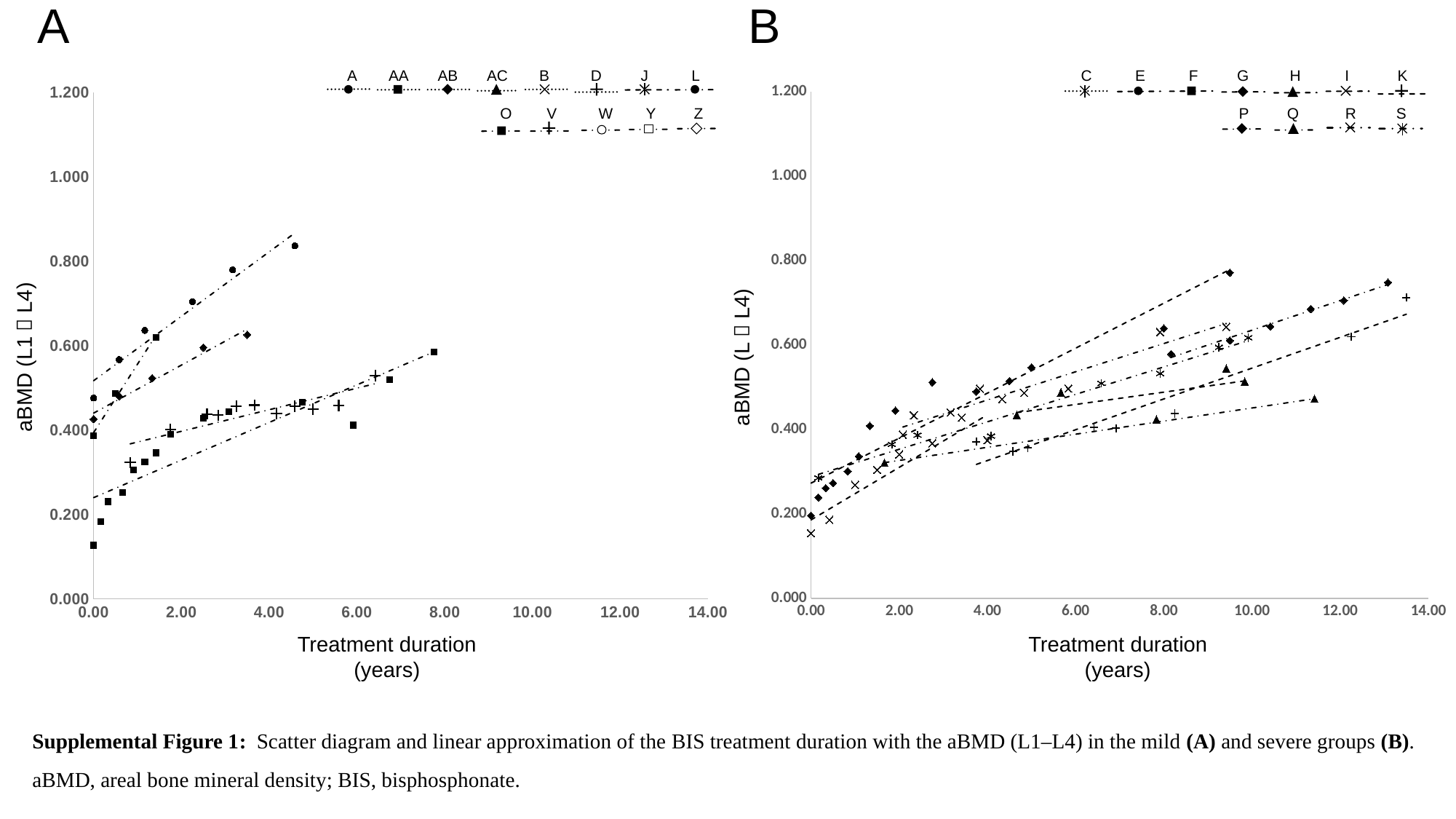

# A
B
### Chart
| Category | C | E | F | G | H | I | K | P | Q | R | S |
|---|---|---|---|---|---|---|---|---|---|---|---|A
AA
AB
AC
B
D
J
L
O
V
W
Y
Z
C
E
F
G
H
I
K
P
Q
R
S
### Chart
| Category | A | AA | AB | AC | B | D | J | L | O | V | W | Y | Z |
|---|---|---|---|---|---|---|---|---|---|---|---|---|---| aBMD (L1－L4)
 aBMD (L－L4)
Treatment duration
(years)
Treatment duration
(years)
Supplemental Figure 1: Scatter diagram and linear approximation of the BIS treatment duration with the aBMD (L1–L4) in the mild (A) and severe groups (B).
aBMD, areal bone mineral density; BIS, bisphosphonate.

## Slide 2
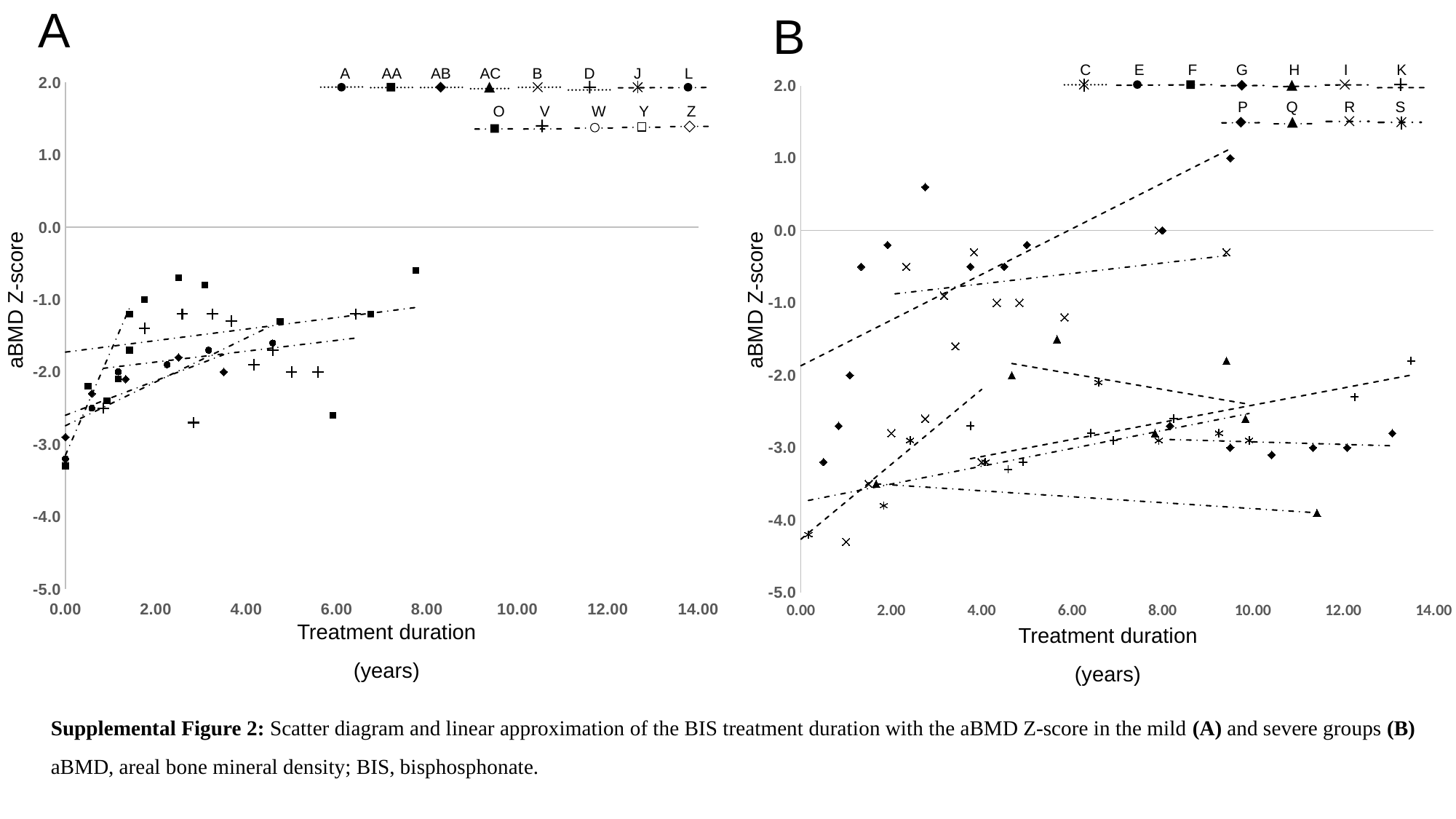

# A
B
### Chart
| Category | A | AA | AB | AC | D | J | L | O | V | W | Y | Z |
|---|---|---|---|---|---|---|---|---|---|---|---|---|
### Chart
| Category | C | E | F | G | H | I | K | P | Q | R | S |
|---|---|---|---|---|---|---|---|---|---|---|---|C
E
F
G
H
I
K
P
Q
R
S
A
AA
AB
AC
B
D
J
L
O
V
W
Y
Z
aBMD Z-score
aBMD Z-score
Treatment duration
(years)
Treatment duration
(years)
Supplemental Figure 2: Scatter diagram and linear approximation of the BIS treatment duration with the aBMD Z-score in the mild (A) and severe groups (B)
aBMD, areal bone mineral density; BIS, bisphosphonate.

## Slide 3
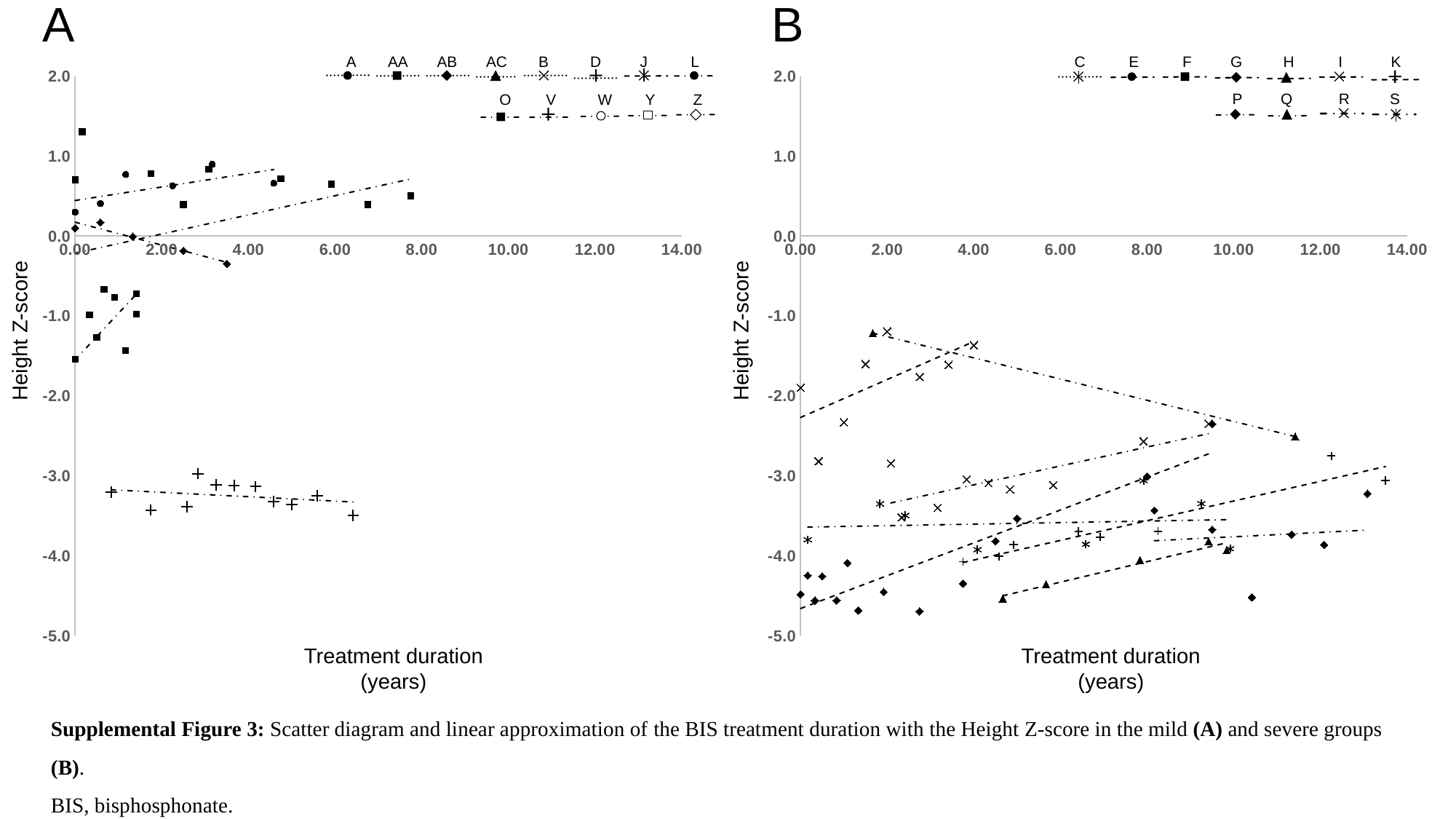

# A
B
C
E
F
G
H
I
K
P
Q
R
S
A
AA
AB
AC
B
D
J
L
O
V
W
Y
Z
### Chart
| Category | A | AA | AB | AC | B | D | J | L | O | V | W | Y | Z |
|---|---|---|---|---|---|---|---|---|---|---|---|---|---|
### Chart
| Category | C | E | F | G | H | I | K | P | Q | R | S |
|---|---|---|---|---|---|---|---|---|---|---|---|Height Z-score
Height Z-score
Treatment duration
(years)
Treatment duration
(years)
Supplemental Figure 3: Scatter diagram and linear approximation of the BIS treatment duration with the Height Z-score in the mild (A) and severe groups (B).
BIS, bisphosphonate.

## Slide 4
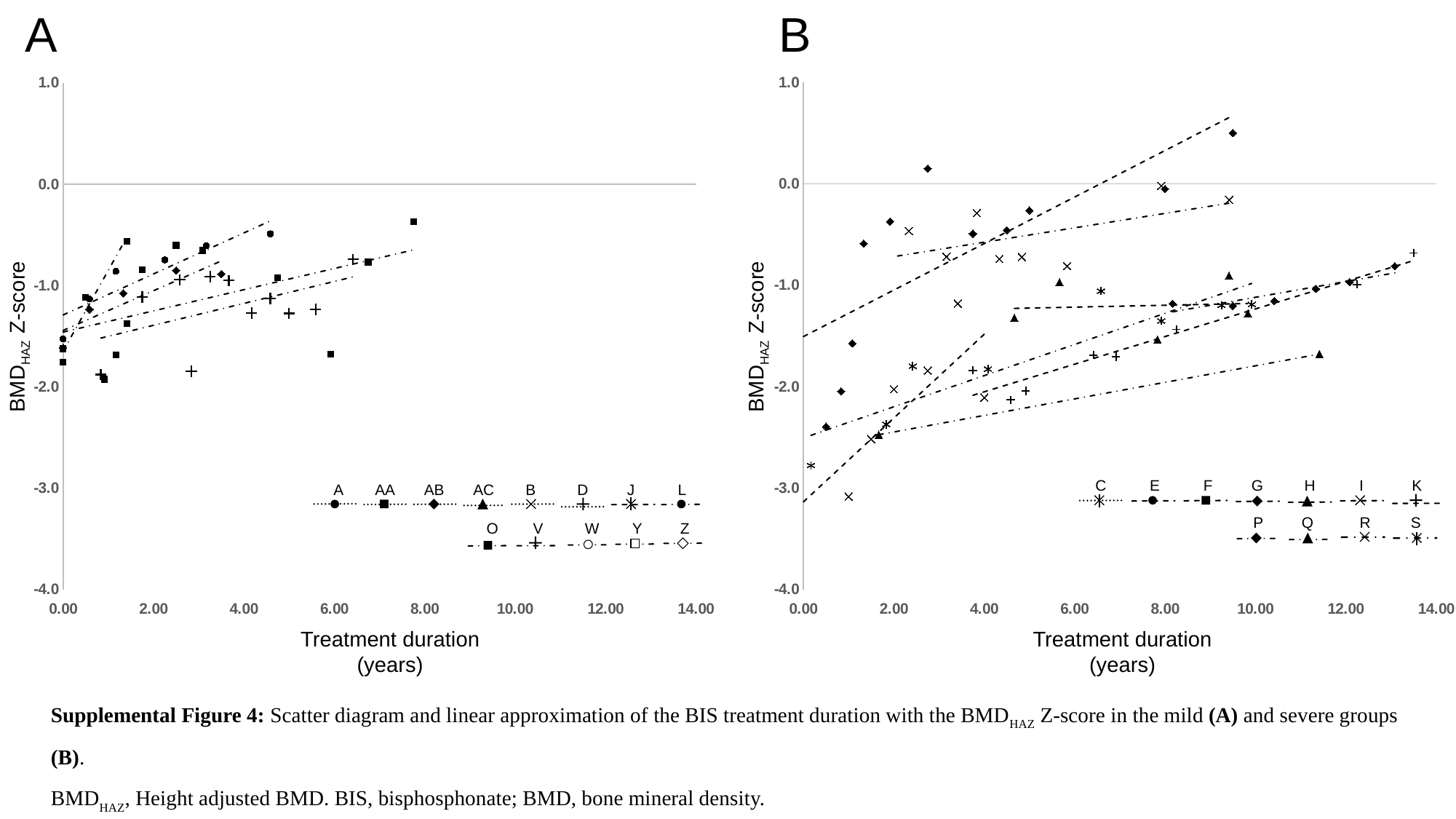

# A
B
### Chart
| Category | C | E | F | G | H | I | K | P | Q | R | S |
|---|---|---|---|---|---|---|---|---|---|---|---|
### Chart
| Category | A | AA | AB | AC | D | J | L | O | V | W | Y | Z |
|---|---|---|---|---|---|---|---|---|---|---|---|---|BMDHAZ Z-score
BMDHAZ Z-score
C
E
F
G
H
I
K
P
Q
R
S
A
AA
AB
AC
B
D
J
L
O
V
W
Y
Z
Treatment duration
(years)
Treatment duration
(years)
Supplemental Figure 4: Scatter diagram and linear approximation of the BIS treatment duration with the BMDHAZ Z-score in the mild (A) and severe groups (B).
BMDHAZ, Height adjusted BMD. BIS, bisphosphonate; BMD, bone mineral density.

## Slide 5
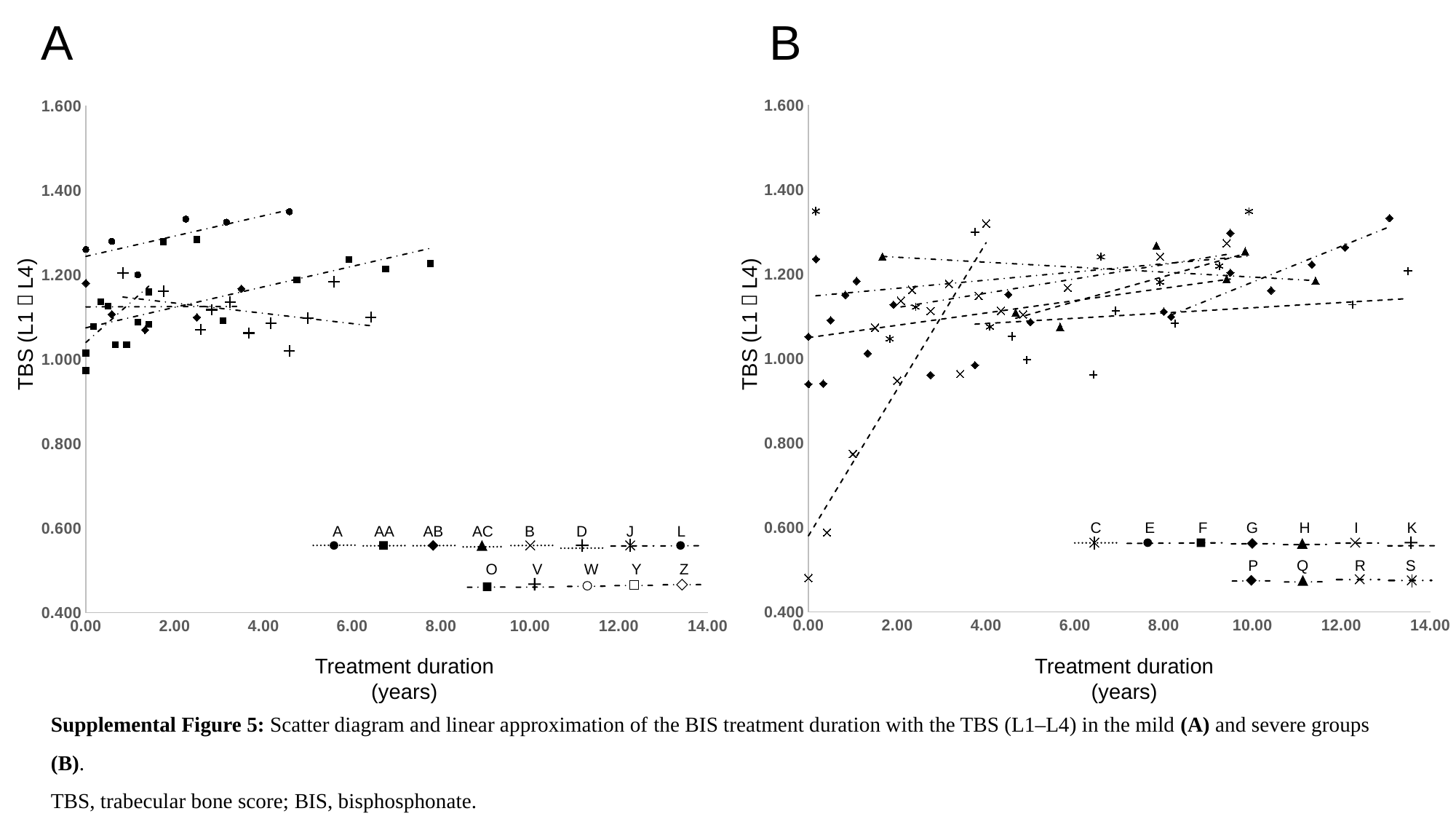

# A
B
### Chart
| Category | C | E | F | G | H | I | K | P | Q | R | S |
|---|---|---|---|---|---|---|---|---|---|---|---|
### Chart
| Category | A | AA | AB | AC | B | D | J | L | O | V | W | Y | Z |
|---|---|---|---|---|---|---|---|---|---|---|---|---|---| TBS (L1－L4)
 TBS (L1－L4)
C
E
F
G
H
I
K
P
Q
R
S
A
AA
AB
AC
B
D
J
L
O
V
W
Y
Z
Treatment duration
(years)
Treatment duration
(years)
Supplemental Figure 5: Scatter diagram and linear approximation of the BIS treatment duration with the TBS (L1–L4) in the mild (A) and severe groups (B).
TBS, trabecular bone score; BIS, bisphosphonate.

## Slide 6
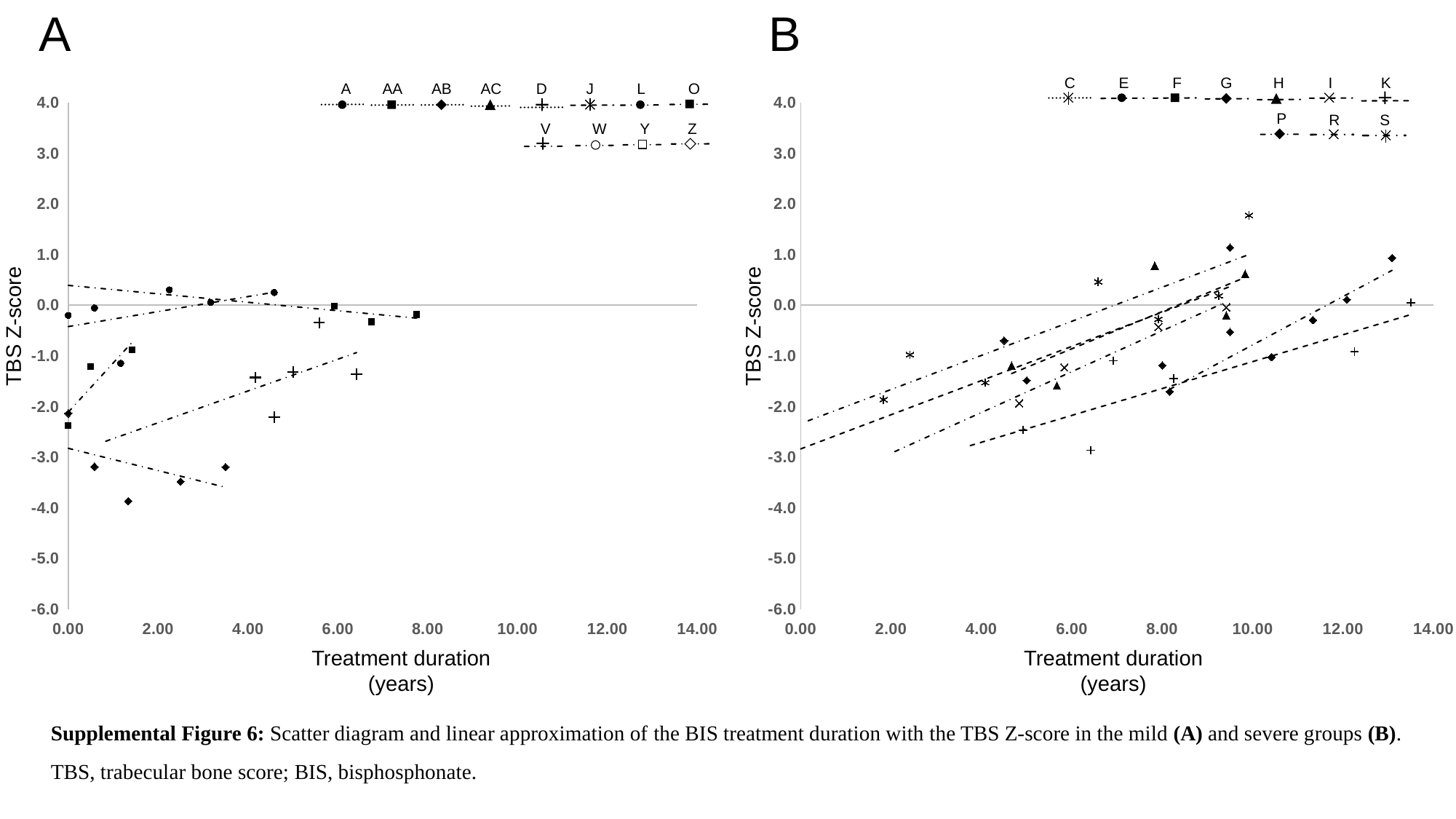

# A
B
C
E
F
G
H
I
K
P
R
S
### Chart
| Category | A | AA | AB | AC | D | J | L | O | V | W | Y | Z |
|---|---|---|---|---|---|---|---|---|---|---|---|---|
### Chart
| Category | C | E | F | G | H | K | P | R | S |
|---|---|---|---|---|---|---|---|---|---|A
AA
AB
AC
D
J
L
O
V
W
Y
Z
TBS Z-score
TBS Z-score
Treatment duration
(years)
Treatment duration
(years)
Supplemental Figure 6: Scatter diagram and linear approximation of the BIS treatment duration with the TBS Z-score in the mild (A) and severe groups (B).
TBS, trabecular bone score; BIS, bisphosphonate.
